# Common misconceptions in crystallography and crystal optics

**DOI:** 10.1107/S1600576725010635

**Published:** 2026-02-01

**Authors:** Lluís Casas

**Affiliations:** a Unitat de Cristallografia i Mineralogia, Departament de Geologia Universitat Autònoma de Barcelona (UAB) Edifici C Cerdanyola del Vallès Catalonia08193 Spain; Wilfrid Laurier University, Waterloo, Ontario, Canada

**Keywords:** misconceptions, X-ray diffraction, cell restrictions, crystal optics, XRD spectra

## Abstract

Did you know that an X-ray diffraction pattern is not a spectrum? However, one in five scientific papers showing X-ray diffraction patterns refers to them as ‘X-ray spectra’. Further, and in contrast to what is stated in many reference books, a triclinic substance could have a cubic-shaped cell. In crystal optics the terminology ‘parallel Nicols’ is almost always misused, and in the frame of polarizing microscope observations zoning is sometimes mistaken for twinning. In this paper, six recurring misconceptions in crystallography, including several related to mineral and crystal optics, are analyzed. Their prevalence is assessed through statistical data on their persistent appearances in peer-reviewed scientific literature. The objective of this study is to promote the correction of these misconceptions in academic communication and teaching. Furthermore, and according to some learning theories, the explanation of well established misconceptions could be used by teachers as an educational resource, offering powerful learning opportunities and contributing to talent development.

## Abstract 2

Six recurrent misconceptions in crystallography, some involving mineral and crystal optics, are analyzed and discussed. The explanation of these and similar misconceptions could serve as educational resources for teachers.

## 1. Introduction

Teaching is a very challenging activity; classrooms are filled with students eager to learn and pursue their aspirations of becoming competent professionals. Students typically do not form a homogeneous group, but rather a diverse community with varying backgrounds, sensibilities, abilities and levels of motivation. This complexity requires educators to employ a range of teaching strategies beyond traditional lectures and demonstrations. Among others, this may include problem-based learning, case studies, a flipped classroom, hands-on experiments and research-based projects. According to constructivist learning theories, mistakes in teaching and learning (Matteucci *et al.*, 2024 ▸) can offer powerful learning opportunities (Rach *et al.*, 2013 ▸). Learning from errors could even be crucial for talent development (Grassinger *et al.*, 2018 ▸).

In science education, there are strong arguments in favor of having instructors who are also active researchers. Universities often promote active scientists as teachers, as their involvement in ongoing research ensures up-to-date knowledge and students benefit from research-informed teaching. Therefore, scientific research, along with teaching, is a central mission of universities, and these two activities are deeply interconnected. Crystallography is no exception to this link between teaching and research. Unfortunately, also in this field several persistent misconceptions are often passed from teachers to students. Some of these are so deeply rooted that they even appear persistently in scientific research papers, further illustrating the strong interplay between the two domains.

Crystallography is a highly interdisciplinary subject that originally developed from the study of the morphology of crystals of naturally occurring minerals. However, soon after the discovery of X-ray diffraction, atomic structure determination became the main research topic in crystallography. Minerals were the first target of structural characterization, but quickly chemists became the main users of crystallographic methods in order to characterize synthetic sub­stances. Besides geology and chemistry, crystallography is currently employed in many other experimental sciences such as physics, biochemistry and materials science (Reventós *et al.*, 2012 ▸). There are not many publications devoted to misconceptions in crystallography. In the field of geology, a review (Francek, 2013 ▸) compiles over 500 misconceptions covering a wide range of topics and organized according to different education levels, including college students and even schoolteachers. However, only four of these listed misconceptions relate to minerals and none relate to crystallography. In the field of chemistry, misconceptions are a recurrent and somewhat more frequently addressed topic. However, crystallographic misconceptions are rarely discussed or often go unnoticed, disguised among other related subjects. In a literature review on misconceptions in chemistry (Suparman *et al.*, 2024 ▸), from 60 reviewed articles none deal with a specific crystallographic issue, and only a few discuss misconceptions of matter/particles and its/their properties. That review focuses on high school teaching. In the book by Barke *et al.* (2009 ▸), historical misuse of the terms molecule and atom in ionic solids like NaCl and KCl is mentioned. The pros and cons of the use of concrete ball-and-stick models for spatial lattice and structure models is one of the few crystallographic topics occasionally mentioned in specialized publications (Barke *et al.*, 2009 ▸; Fensham & Kass, 1988 ▸). The lack of articles specifically devoted to misconceptions in crystallography is possibly due to the high degree of specialization of the topic, which is generally not covered in education below the college level. Nevertheless, there are two published papers on the subject, one (Brock & Lingafelter, 1980 ▸) highlighting five serious misconceptions about crystal lattices and crystal sym­metry that appear recurrently in general and physical chemistry textbooks, and another (Prasad, 2016 ▸) discussing errors in *The Feynman lectures on physics* related to the concept of symmetry. The present contribution aims to explore further misconceptions within the domain of crystallography and crystal optics, a topic intimately related to mineralogy.

## 2. Methods

Six common misconceptions are explored in detail. These were identified through direct personal experience (first as a student and later as a lecturer). They were selected after confirming that they are deeply rooted not only among students but also within the academic community. I have encountered them in student assessments, as well as at scientific conferences and in published research papers. For each selected misconception, the detailed problem is first explained, including an analysis of the reasoning that underlies it. This step is essential for understanding why these misconceptions are so persistent, and it also serves as a foundation for developing arguments that can help both scientists and educators recognize the misconception and potentially use it as a teaching tool.

For those misconceptions that are easily traceable, the SCIDIR Search API from the ScienceDirect database (https://www.sciencedirect.com/) was used to quantify the number of annual occurrences containing the misconception. These searches allow for examination of key details, such as the first appearance of the misconception in scientific literature and its evolution over time. Depending on the results obtained, certain statistical analyses were conducted, for instance, the percentage of incorrect uses of the concept relative to its total number of citations. This type of information can help in clarifying the underlying reasons for the persistence of these misconceptions.

## 3. Results and discussion

The six examples are discussed separately and in detail below. They are summarized in Table 1 ▸ so that interested readers can quickly identify those that attract their attention and then explore the corresponding subsections in more depth. The selected misconceptions have been highlighted because of their significance and recurrence in indexed peer-reviewed scientific papers.

### 3.1. Cell restrictions

A very common misconception in crystallography, already highlighted by Brock & Lingafelter (1980 ▸), concerns a fundamental notion, namely the unit cell and its possible shapes. It is well known that there are six crystal families, and quite often these are described using their corresponding unit cells. Very often the restrictions are presented for the seven crystal systems, although the restrictions of trigonal and hexagonal systems are identical and that is why these two systems are grouped within the hexagonal crystal family. Regardless of whether one is referring to crystal systems or families, it is very common to find the presentation of the cubic cell indicating its orthogonality (α = β = γ = 90°) and the equivalence between the cell axes (*a* = *b* = *c*), and analogously the triclinic cell is often defined using inequalities (*a* ≠ *b* ≠ *c* and α ≠ β ≠ γ ≠ 90°). These inequalities, and any other regarding other crystal families, are strictly speaking incorrect as there is nothing that prevents a coincidence between the modulus of the cell vector or their angles, and the angle could be coincidentally square. Even generative AI tools describe the crystal metrics using inequalities. The misconception is detailed in an excellent introductory textbook [see Section 1.8 of Hoffmann (2020 ▸)] where it is described as a ‘common though wrong overview of the metric of the seven crystal systems’.

It is not easy to find scientific papers explicitly revealing this misconception, and that is in part because the definition of a crystal family (or its corresponding system) is not commonly found in regular papers. However, when the definition is explicit, we can detect the misconception (*e.g.* Nageeb *et al.*, 2024 ▸). In contrast, it is not difficult to spot this conceptual error in crystallography textbooks (Allewell & Trikha, 1995 ▸; Allen *et al.*, 2012 ▸; Perkins, 2020 ▸), although many others do not use inequalities and define the different cells by just stating the cell restrictions. Also, in many online teaching materials about crystallography and powder diffraction the error is recurrent, even in web pages hosted by universities.

This misconception could be perceived as a minor issue, but it often reflects a deeper misunderstanding. Many students, and even teachers, believe that the shape of the unit cell determines the crystal system of a crystalline solid. However, this is not the case. In fact, for a given periodic arrangement, there are infinitely many possible unit cells with varying morphologies. It is the symmetry of the structure, not the shape of the unit cell, that ultimately determines the crystal system. The conventional unit cell is chosen to reflect the full symmetry of the crystal system, and this is why centered cells are sometimes preferred to primitive cells. On the opposite side of the question, very occasionally a cell morphology could suggest a much higher symmetry than the one determined by its atomic content. For instance, NaKZnP_2_O_7_ is a monoclinic phosphate (space group *P*2_1_/*n*) with a unit cell with orthogonal angles (Shepelev *et al.*, 2006 ▸). The orthogonality of the cell could suggest that this phosphate belongs to the orthorhombic crystal system, but the symmetry of its atomic content determines that it belongs to the monoclinic system instead. Examples like this real structure should be used by teachers to show that the inequalities are inadequate to define the metric of the crystal systems; instead of the inequality (*e.g.a* ≠ *b*) they should just mention that there are no constraints (*e.g.a*, *b* independent). Regarding the angles, similar inequalities (*e.g.* α ≠ β ≠ γ) should be avoided. The angles can assume any value but, strictly speaking, they are not independent, and some combinations can result in impossible cell volume values (Foadi & Evans, 2011 ▸).

### 3.2. Trigonal (crystal system) versus rhombohedral (lattice system), and the role of hexagonal

There is some confusion regarding hexagonal, trigonal and rhombohedral terms and the International Union of Crystallography (IUCr) is the authorized institution to clarify the subject (Souvignier, 2016 ▸). There are seven crystal systems, among them the trigonal and hexagonal systems. Each crystal system contains a number of geometric crystal classes which have a one-to-one correspondence with the 32 point symmetry groups. Trigonal and hexagonal point groups have a lot in common: for instance, all of them share a threefold rotation axis. The hexagonal groups have either a sixfold rotation axis or a sixfold roto-inversion axis, and both types of axes contain the threefold rotation axis. In fact, a sixfold roto-inversion axis is equivalent to a threefold rotation axis combined with a mirror plane perpendicular to it. Trigonal and hexagonal crystal systems are grouped in the hexagonal crystal family (one of the six existing crystal families). The hexagonal family contains two lattice systems, called hexagonal (*P*) and rhombohedral (*R*). It would be erroneous to use the terms trigonal or rhombohedral to refer to a crystal family, but these appear occasionally in published papers (Chernyavsky *et al.*, 2022 ▸; Barbosa *et al.*, 2020 ▸; Kothari & Kanchan, 2021 ▸). To further confuse matters, the rhombohedral lattice system can be described using hexagonal (*a* = *b*, α = β = 90°, γ = 120°) or rhombohedral (*a* = *b* = *c*, α = β = γ) settings; the first implies a centered cell and the latter a primitive cell. From the 25 trigonal space groups, seven are assigned to the rhombohedral (*R*) lattice system and 18 to the hexagonal lattice system (*P*); the corresponding space groups are listed in Table 1.3.4.3 in *International tables for crystallography*, Vol. *A* (Souvignier, 2016 ▸).

**Figure 1:**
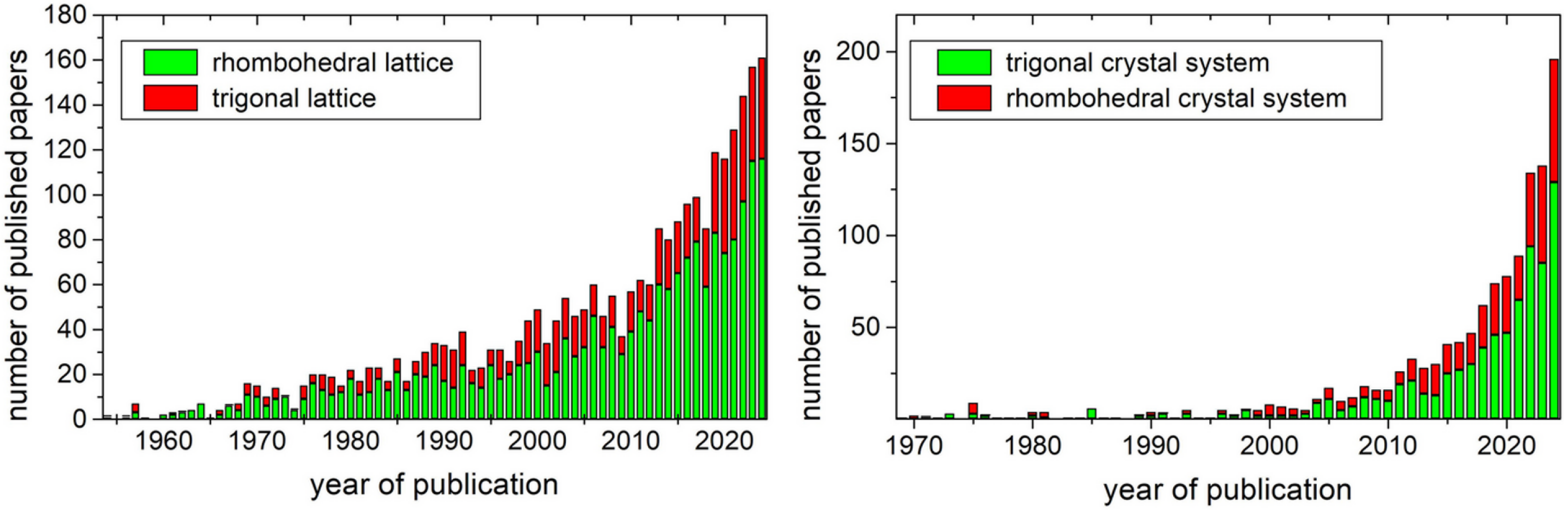
Left, stacked column plot showing the number of published papers containing either the correct term ‘rhombohedral lattice’ (green) or the inaccurate ‘trigonal lattice’ (red). Right, similar stacked column plot with the papers using the correct term ‘trigonal crystal system’ (green) or the inaccurate ‘rhombohedral crystal system’ (red). Data obtained using SCIDIR search API.

Note that the adjective trigonal refers to a crystal system whereas rhombohedral refers to a lattice system or a type of cell. Therefore, it is not formally correct to use the names ‘trigonal lattice’ or ‘rhombohedral crystal system’. However, these names are regularly spotted in scientific papers (Fig. 1 ▸). The distinction between trigonal and rhombohedral is not new – it was formalized in the first of the series that was to become *International tables for crystallography* published in the 1930s. The number of papers mentioning the terms trigonal or rhombohedral is growing year on year, and the misconception is so widespread that the faulty ‘rhombohedral crystal system’ remains at about 35% of the total yearly mentions including ‘rhombohedral’ and ‘trigonal crystal system’. A similar percentage can be found when computing the mentions of the faulty ‘trigonal lattice’ over the total mentions of ‘rhombohedral’ and ‘trigonal lattice’. Such recurrence indicates that many authors might regard the terms rhombohedral and trigonal as synonyms. However, researchers should strive to use the terms correctly and in accordance with the definitions provided by the IUCr, and teachers should do the same in their lectures on crystallography.

### 3.3. XRD, a spectroscopic technique?

Spectroscopic techniques (Skoog *et al.*, 2017 ▸) are those measuring energy changes (absorption/emission) resulting from the interaction between photons and a sample of matter. These energy changes usually involve interaction with electrons, chemical bonds or atomic nuclei. Depending on the type of interaction, spectroscopy can be classified as electronic (*e.g.* UV–Vis, X-ray fluorescence or X-ray absorption spectroscopy), vibrational (*e.g.* Raman and infrared spectroscopies) or nuclear (*e.g.* nuclear magnetic resonance or Mössbauer spectroscopy). Each spectroscopic technique provides specific information about the sample, such as chemical composition, oxidation states, bonds, functional groups *etc*. The results are typically presented in a spectrum, a plot showing intensity (or simply measured counts) as a function of a characteristic related to the inelastic interaction, such as wavelength, frequency or energy.

In contrast to spectroscopic techniques, in X-ray diffraction (XRD) the radiation interacts elastically with the sample, *i.e.* the wavelength (and the related energy) remains unchanged. Diffraction is an interference phenomenon that arises when radiation waves deviate around atoms, which act as obstacles. In crystalline materials, the regular atomic arrangement produces constructive interference in specific directions, resulting in distinct diffraction peaks emerging from a noisy background. These directions and their associated intensities carry valuable information about the structural arrangement of the atoms. Despite the similarities with some electron spectroscopies, XRD is not a spectroscopic technique and the corresponding results, usually presented in the form of a plot showing intensity (or counts) as a function of the scattering angle (2θ), cannot be named XRD spectra; the correct name for the plot is a diffractogram (or diffraction pattern).

Certain specialized techniques, such as energy-dispersive XRD (EDXRD) or time-of-flight (TOF) neutron diffraction, employ polychromatic radiation rather than the monochromatic beams used in conventional XRD. This can lead to resulting data being described as spectra since they display radiation intensity as a function of energy. However, even in these cases, the underlying physical principle remains Bragg diffraction, not an interaction involving energy transitions as in *sensu stricto* spectroscopic techniques.

**Figure 2:**
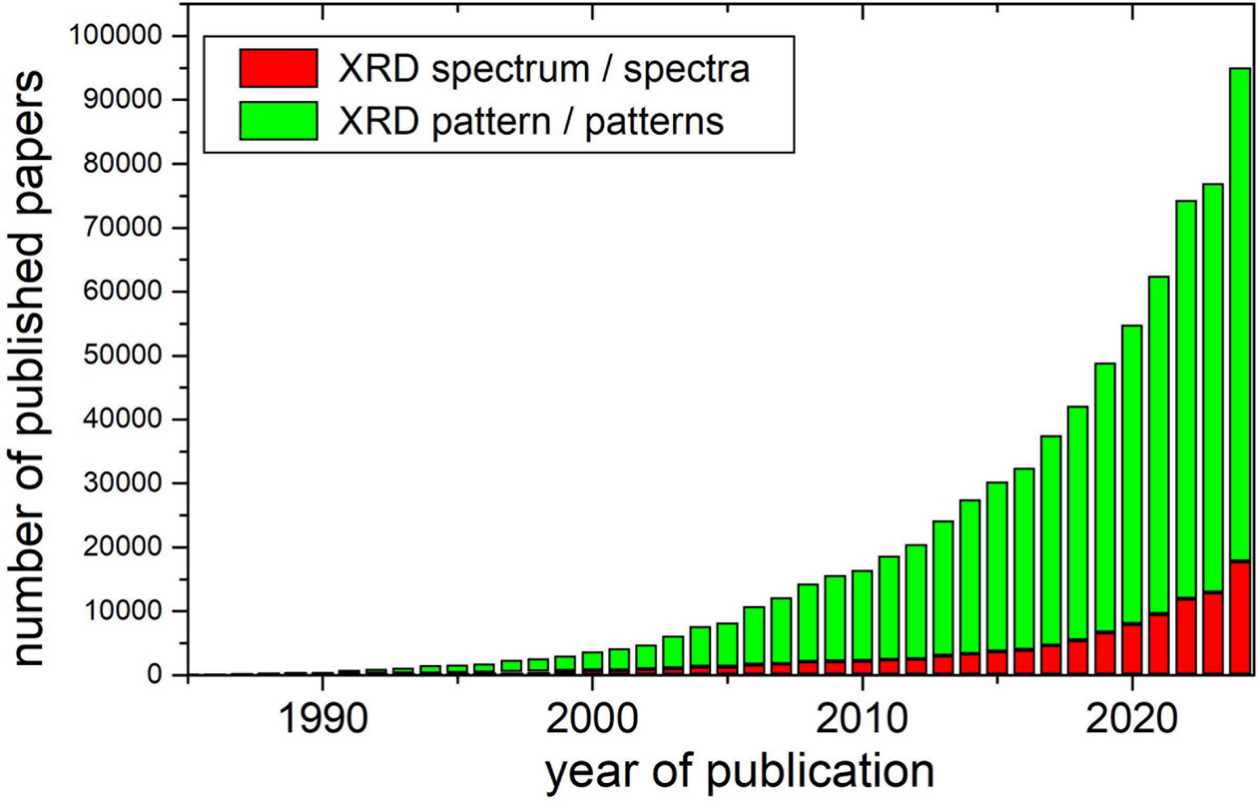
Stacked column plot showing the number of published papers containing either the correct term ‘XRD pattern’ (green) or the inaccurate ‘XRD spectrum’ (red). Data obtained using SCIDIR search API.

Some fundamental books on XRD already point out the common misinterpretation of XRD patterns as spectra (Iwashita, 2016 ▸). However, the number of papers using the faulty term ‘XRD spectrum’ is substantial and it is continuously increasing (Fig. 2 ▸). In 2024 there were 17723 published papers mentioning ‘XRD spectrum’ or ‘XRD spectra’, which is ∼19% of the total published papers containing either ‘XRD spectrum/a’ or ‘XRD pattern/s’ in the text. In fact, the percentage of papers referring to XRD spectra over the total number of papers dealing with XRD seems to have stabilized at around 20% since 1990. Generative AI tools like ChatGPT or Gemini can occasionally refer to XRD patterns as spectra, and this is due to the huge number of papers using this faulty terminology. It is the task of specialists to correct colleagues using the erroneous term in their scientific and academic communications.

### 3.4. Alloy versus compound

Within the fields of crystallography and mineralogy, but also in other solid-state fields like materials science, it is not rare to hear (and read) the terms ‘alloy’ and ‘intermetallic compound’ interchanged and misused. An intermetallic compound refers to any class of substance composed of definite proportions of two or more metals, rather than continuously variable proportions (as in solid solutions). A solid solution involving metals can be also named a homogeneous or monophase alloy (as in brass, a copper and zinc alloy). Therefore, there is an inherent disorder in the case of homogeneous alloys, *i.e.* two or more chemical elements (or even a vacancy) are randomly distributed on a given crystallographic site. In contrast, compounds lack this kind of disorder as every element has definite positions not shared with other elements. In terms of properties, those of alloys shift smoothly from those of their pure constituents whilst those of intermetallic compounds differ markedly from those of their constituents.

The ambiguity between alloys and compounds is possibly inextricably linked to the use of the term alloy not only for homogeneous substances but also for substances with a microstructure made of two or more phases. These multiphase or heterogeneous alloys are in fact made of compounds with definite proportions (*i.e.* there is not inherent crystallographic disorder in them). A clear example of these heterogeneous alloys is pearlite, which is a microstructure in carbon steels that consists of alternate layers containing ferrite (body-centered cubic Fe) and cementite (Fe_3_C). To make things more complex, pearlite is the product of low-temperature decomposition of austenite, which has a face-centered cubic structure containing Fe and C and is a monophase alloy.

**Figure 3:**
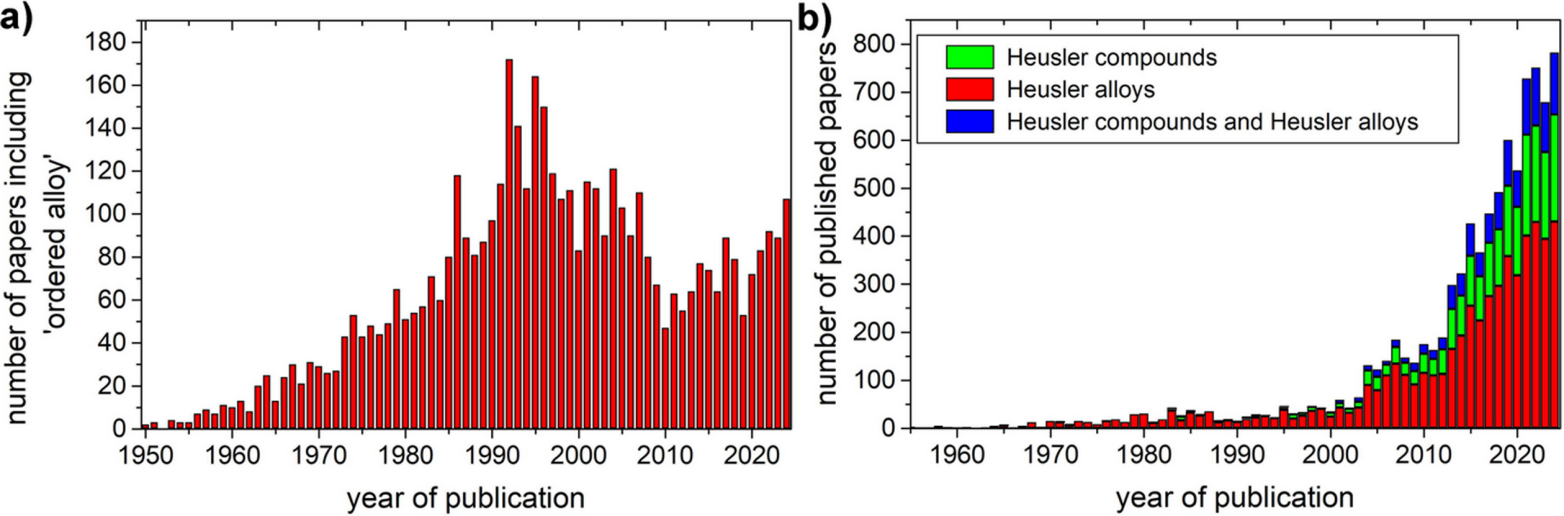
(*a*) Histogram showing the number of published papers containing the term ‘ordered alloy’. (*b*) Stacked column plot with the papers using the correct term ‘Heusler compound/s’ (green), the inaccurate ‘Heusler alloy/s’ (red) or those combining both terms (blue). Data obtained using SCIDIR search API.

Another factor contributing to the confusion between the terms intermetallic compounds and alloys is the broader definition of ‘solid solution’ that appears in the fields of materials science and metallurgy; this includes ‘ordered’ solid solutions formed by mixtures of two or more elements that occupy specific sites in the crystal structure. These ‘ordered’ solid solutions are nonsense from the crystallographic perspective and they should simply be called compounds (intermetallic compounds in the event of forming structures using two or more metals). The ambiguity seems to be restricted to intermetallic substances, as no-one would dare to refer to minerals like pyrite (FeS_2_), magnetite (Fe_3_O_4_) or even ice (H_2_O) as ordered solid solutions. In contrast, ordered structures like Fe_3_Al, Ni_3_Al, L1_0_-AuCu or β-CuZn (ordered brass) are often considered homogeneous alloys. In fact, the term ‘ordered alloy’ appears regularly in the scientific literature [Fig. 3 ▸(*a*)].

Even admitting that the official definition of alloy allows the inclusion of the heterogeneous alloys and the homogeneous ordered solid solutions, there are still some recognized misuses of the term alloy in the literature. In the specific field of magnetic materials, the Heusler compounds (discovered in 1903) are often referred to as Heusler alloys, but some authors acknowledge that the term intermetallic compound is more appropriate (Graf *et al.*, 2011 ▸) as these compounds have no disorder. However, the use of alloy to refer to these fully ordered compounds is well established. In Fig. 3 ▸(*b*) we see that the presence of papers using the name ‘Heusler compounds’ is slowly advancing but ‘Heusler alloys’ is still the predominant (∼66%) formula to refer to them. Moreover, there are a significant number of papers that combine both names (Heusler alloys and compounds), which could contribute to perpetuate the confusion.

### 3.5. Parallel Nicols

**Figure 4:**
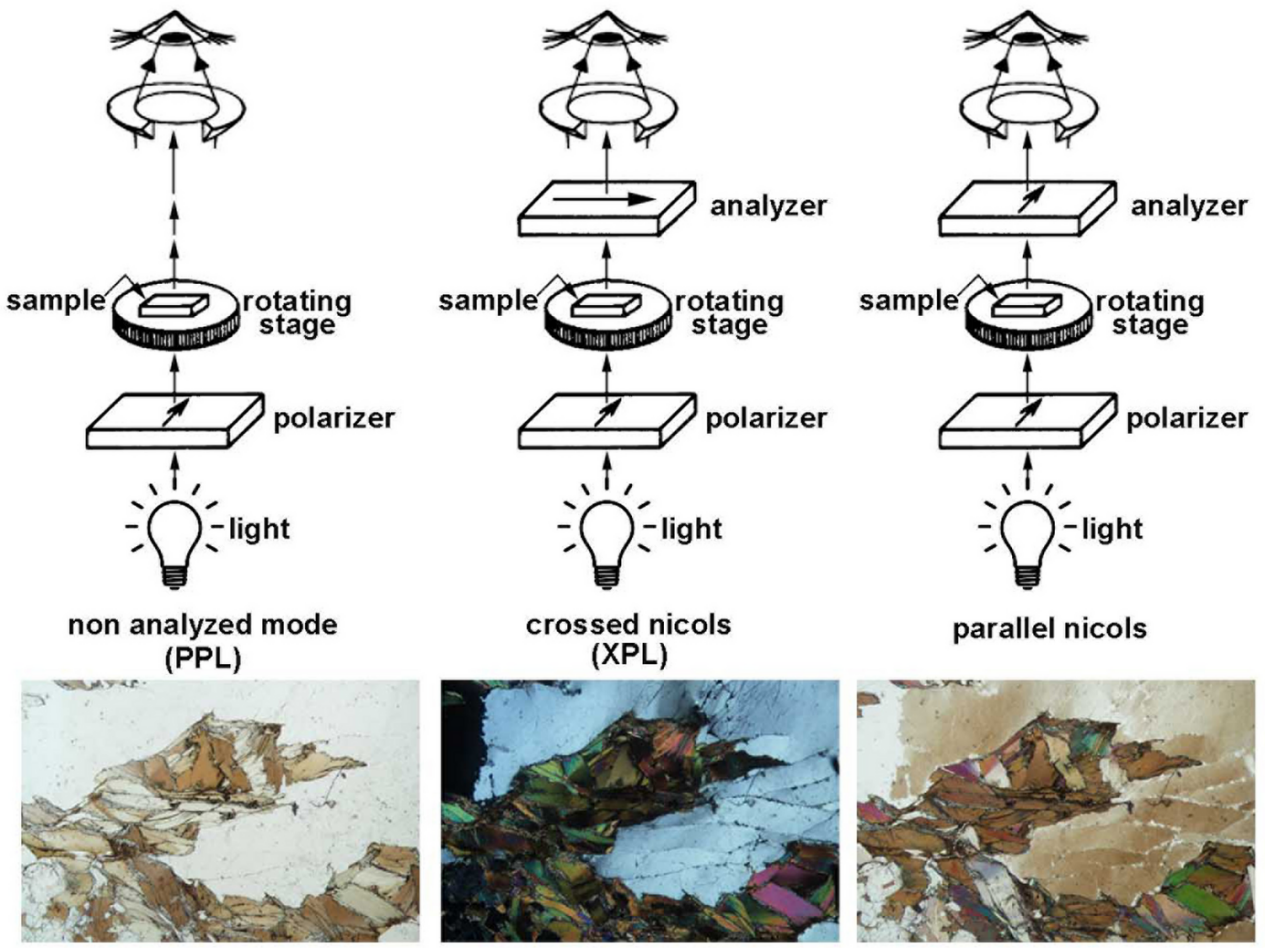
Schemes of three types of configuration in a petrographic microscope; the arrows on polarizers indicate their polarization directions. Left, plane polarized light (PPL); middle, crossed Nicols (XPL); right, parallel Nicols. Below each scheme is the resulting petrographic image of a rock containing quartz and biotite.

Petrographic microscopes make use of polarizers to observe minerals and rocks prepared as thin sections. An alternative name for the polarizer is nicol, often capitalized, in reference to the Nicol prism, a type of polarizer made from a crystal of calcite crystal, invented in 1828 by Scottish geologist William Nicol. Nowadays, most microscopes use a different type of polarizer, which is cheaper and made of polymer (sheet polarizers). However, the term Nicol or nicol persists, and it is used to describe two different microscope settings. Characteristically, petrographic microscopes use two Nicol prisms mounted co-axially: a first Nicol produces the plane polarized light that enters the thin section under observation and this filter is called simply the polarizer, while a second Nicol prism, the analyzer, analyzes the incoming light from the sample. Typically, the polarization directions of the two Nicols are mutually perpendicular. Thus, without a sample in the optical path the light from the polarizer cannot be transmitted through the analyzer and the intensity of the field of view should be zero. This setting is known as crossed Nicols and is often abbreviated to XPL (cross-polarized light). In contrast, when the polarization directions of the two Nicols are parallel, the setting is called ‘parallel Nicols’, and then, without a sample in the optical path, the analyzer does not intercept the light and the intensity of the field of view is maximum. In both settings (crossed and parallel Nicols), when a non-cubic mineral is under observation, this will display its characteristic interference colors (Fig. 4 ▸). For a given mineral section, the interference colors observed in crossed and parallel Nicols constitute a complementary chromatic pair [see *e.g.* Fig. 4.13 of Houck & Siegel (2010 ▸) and Fig. 4 ▸].

Despite the clear definitions of the two settings, easily found in basic materials on optical microscopy (Olympus Corporation, https://www.yumpu.com/en/document/read/17552622/basics-of-polarizing-microscopy-olympus), it appears that the term ‘parallel Nicols’ is often wrongly used by both academics and students. Even in research papers the term is often misused and confused with the PPL (plane polarized light) setting. In PPL mode only a polarizer is required; the analyzer is therefore removed from the optical path (Fig. 4 ▸). PPL mode is used to observe the true colors of the minerals (not the interference colors).

**Figure 5:**
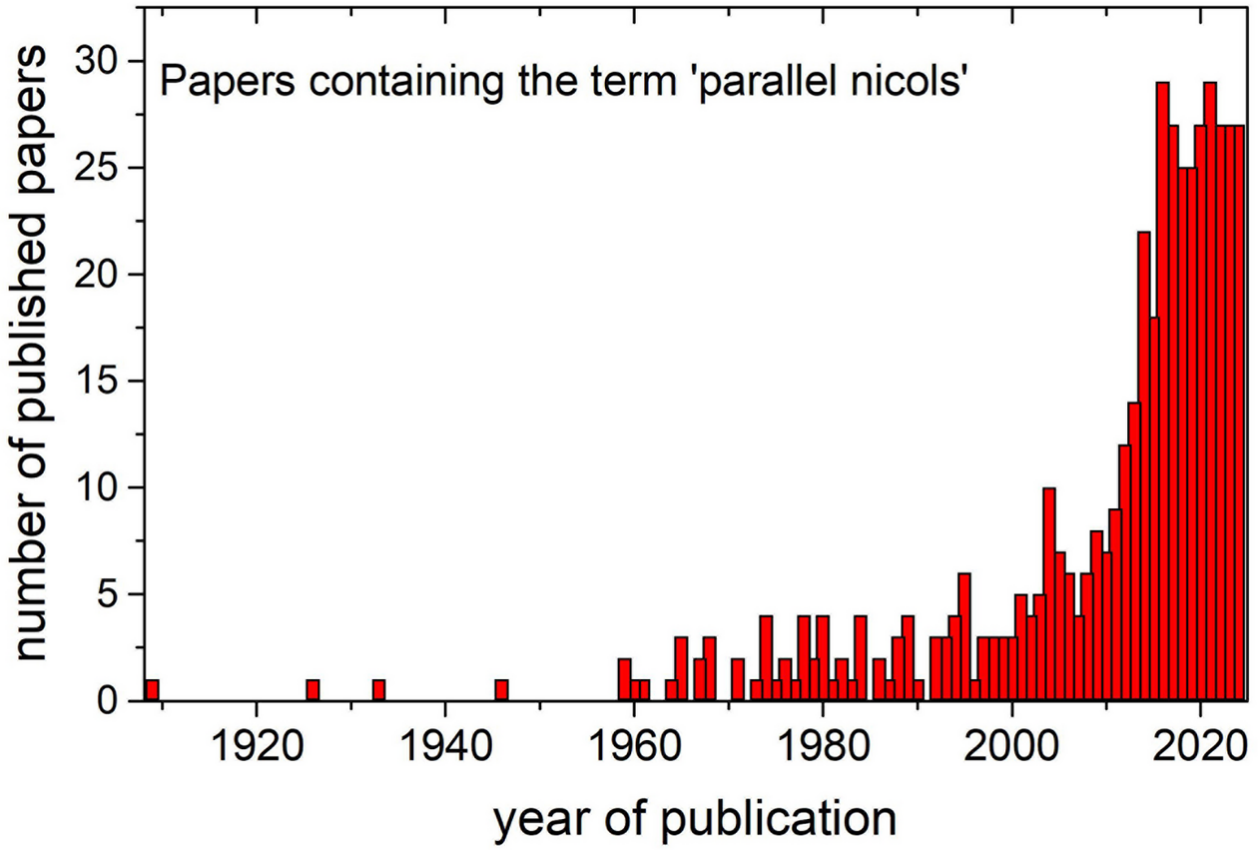
Histogram showing the number of published papers containing the term ‘parallel Nicols’. Data obtained using SCIDIR search API.

**Figure 6:**
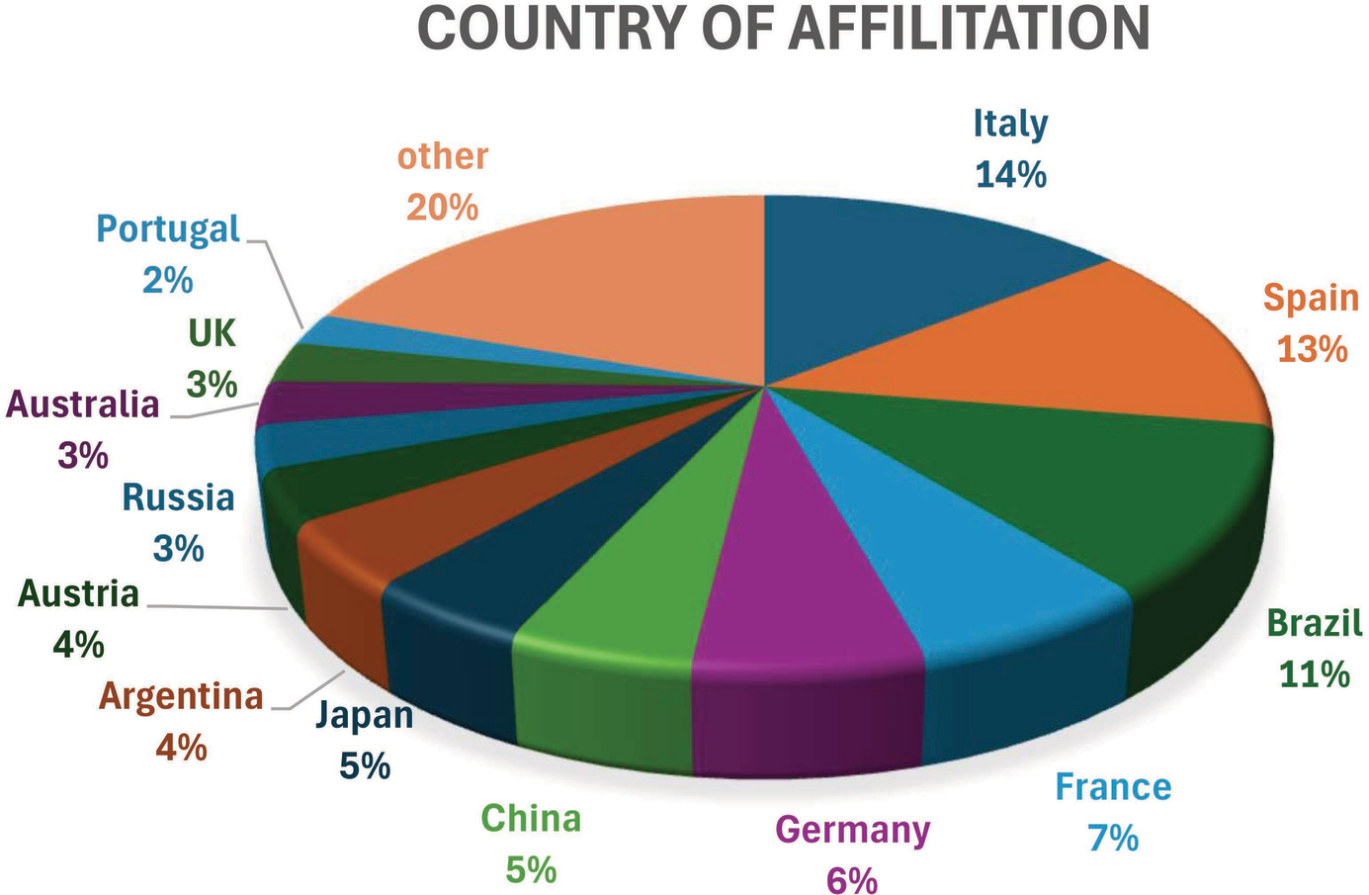
Pie chart showing the distribution of countries represented in the authors’ affiliations of papers (published between 1909 and 2024) that use the term ‘parallel Nicols’.

Checking the scientific literature, the first paper using the term ‘parallel Nicols’ dates from 1909 (Evans, 1909 ▸), and until the 1960s the term only appears sporadically. Then the term can be regularly found almost every year at least in one paper. Around the 2010s the number of papers including the term ‘parallel Nicols’ increases abruptly from ∼10 to ∼25, and in recent years this number has stabilized at around 20–30 cites (Fig. 5 ▸). In the first papers mentioning ‘parallel Nicols’ the compound term is mostly correctly used. However, in some of them (*e.g.* Kargin *et al.*, 1964 ▸; Möller *et al.*, 1974 ▸) the term appears in the figure caption of black-and-white micrograph pictures, and it is hard to see if the image has been really obtained in parallel Nicols or simply in the non-analyzed PPL. In particular, when the term is used in combination with other images taken in crossed Nicols, there appear reasonable doubts about the correctness of the ‘parallel Nicols’ specification (Schneider & Majdič, 1980 ▸). The term was possibly already misused in some papers from the 1980s (Kienast & Rangin, 1982 ▸; Larson & Tullborg, 1984 ▸), but again the black-and-white images do not allow us to discern the presence or not of interference colors in the images. In structural geology papers dealing with quartz and phyllosilicates (Casas, 1986 ▸; Román-Berdiel *et al.*, 2004 ▸) and despite the persistence of black-and-white images, the absence of contrast between grains of different orientation indicates that the supposedly ‘parallel Nicols’ are in fact non-analyzed (PPL) micrographs. In later papers including high-quality color images (Freire-Lista *et al.*, 2022 ▸; de Oliveira *et al.*, 2014 ▸; Armas *et al.*, 2014 ▸), it is easy to notice that the term ‘parallel Nicols’ is effectively misused, and this is the common situation in the presently published papers except for a few exceptions (*e.g.* Sanders *et al.*, 2018 ▸). A systematic survey over the 27 papers published in 2023 mentioning ‘parallel Nicols’ reveals that the term is probably misused in four of them and clearly misused in the other 23 [see *e.g.* Fig. 5 of Gibert *et al.* (2023 ▸)]. A thorough examination of the authors’ affiliations reveals that the issue concerns mainly researchers from non-English-speaking countries (see Fig. 6 ▸). Italian, Spanish and Brazilian authors appear the most inclined to use the term, often misusing it, especially in recent publications. Native English speakers, on the other hand, may be more aware of the meaning of the standard acronym PPL and are therefore less likely to confuse it with the term ‘parallel Nicols’.

The origin of the faulty use of ‘parallel Nicols’ can be in part attributed to the use of ambiguous black-and-white pictures in early scientific papers. Effectively, distinction between non-analyzed PPL images and parallel Nicols micrographs can be difficult. The true ‘parallel Nicols’ setting is nowadays hardly used by researchers and teachers, but the term has continued to be misused as a counterpart of the widely used ‘crossed Nicols’. Researchers and particularly teachers should contribute to change this situation by using the pair PPL (plane polarized light) and XPL (cross-polarized light).

### 3.6. Hourglass twinning or zoning

**Figure 7:**
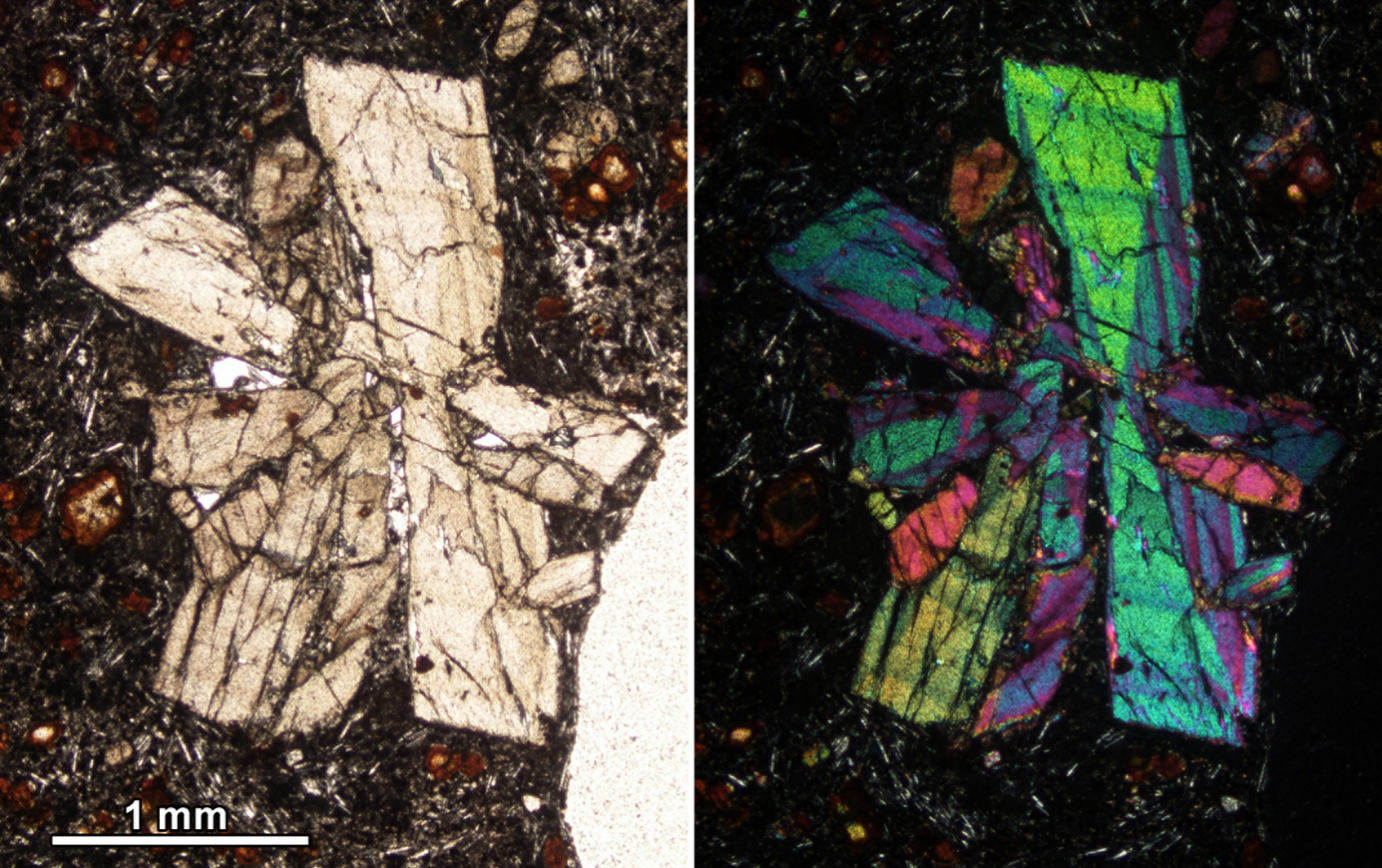
PPL (left) and XPL (right) images of an aggregate of clinopyroxene crystals displaying hourglass zoning.

In the context of petrographic descriptions, it is common to hear informal descriptions of twinned crystals with popular names, for instance ‘fishtail’ (for gypsum, herderite or epididymite) or ‘cross-shaped’ (staurolite, arsenopyrite) or ‘iron-cross’ (pyrite) twins. Another usual name is the ‘hourglass twin’, particularly to refer to clinopyroxenes and tourmaline. This appears in XPL as a section divided into four (sometimes six or eight) wedge-shaped sectors with different interference colors that radiate from the center of the section forming a symmetric cross resembling an hourglass (Fig. 7 ▸).

In the case of both clinopyroxenes and tourmaline, these are not really twinned crystals but single crystal sections with a compositional zoning. In clinopyroxenes like diopside (CaMgSi_2_O_6_) these are commonly Al–Ti–Na-rich sectors and Mg–Ca-rich sectors (Welsch *et al.*, 2015 ▸). In tourmaline the sectors generally divide into Fe–Ti–Ca–Na–F-rich and Mg–Li-rich and they are already visible using non-analyzed PPL (Hinsberg *et al.*, 2006 ▸). The zoning is produced by growth in non-equilibrium conditions (MacDonald *et al.*, 2024 ▸). The elements that do not incorporate efficiently into the structure are pushed into the fast-growing faces whilst the rest (*e.g.* Mg and Ca in clinopyroxenes) appear enriched in the slow-growing faces.

The confusion between twinned crystals and a zoned crystal may seem unimportant. After all, it is quite understandable that under a petrographic microscope, especially using XPL, the boundaries between sectors in this type of zoning can be mistaken for twin planes. However, from an educational perspective, it is important to emphasize the distinction. Clarifying the difference allows for a more accurate exploration of both twinning and zoning, along with their respective genetic implications in crystal growth.

**Figure 8:**
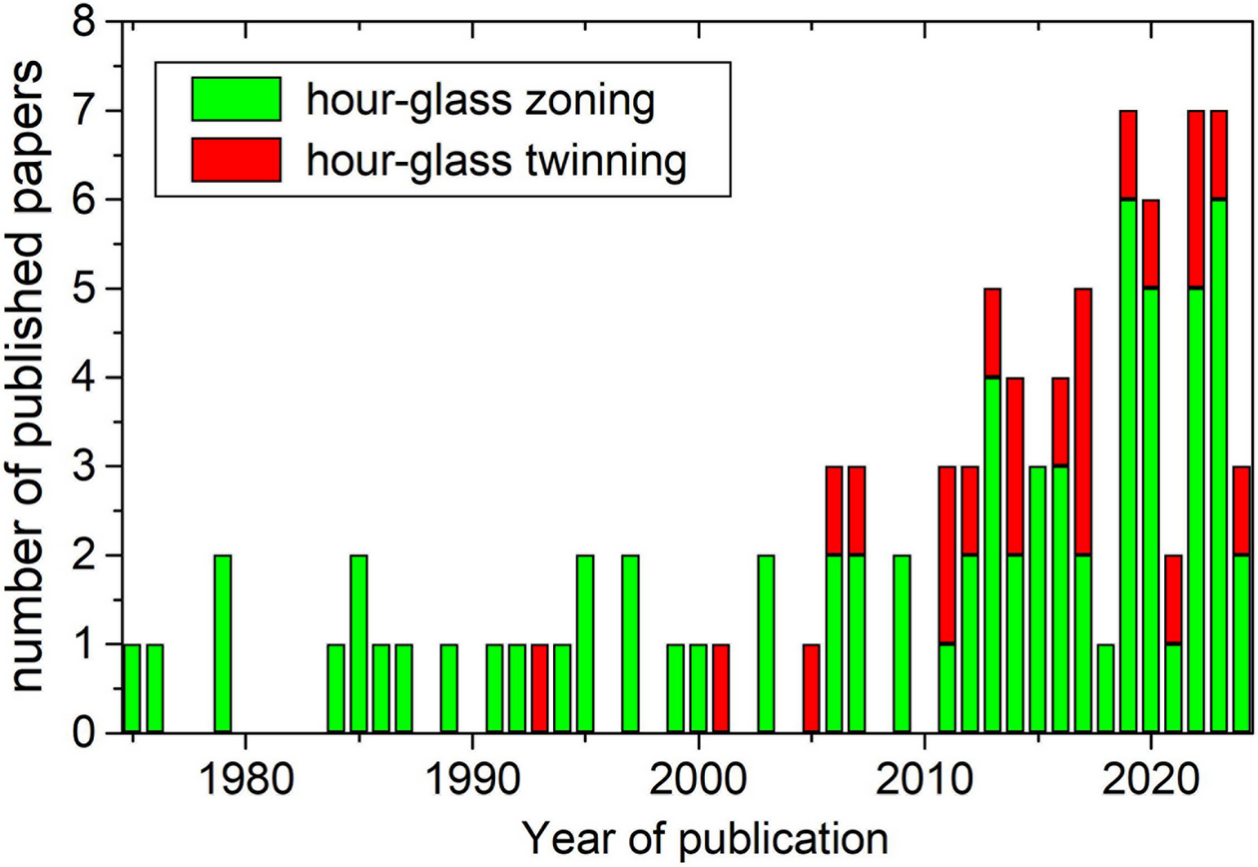
Stacked column plot showing the number of published papers containing either the correct term ‘hourglass zoning’ (green) or the inaccurate ‘hourglass twinning’ (red). Data obtained using SCIDIR search API.

In the scientific literature it is not very common to find this confusion (Fig. 8 ▸) and there are not many papers on the subject. From 1993 only 22 papers use erroneously the term twinning to refer to this zoning (*e.g.* dos Santos *et al.*, 2022 ▸; Maia *et al.*, 2021 ▸; Fazlnia, 2019 ▸; Belkacim *et al.*, 2017 ▸; Carracedo-Sánchez *et al.*, 2016 ▸), with 59 papers using the correct terminology. Still, the proportion of papers referring erroneously to this particular zoning as twinning (∼1/3) shows that this is another well established misconception among many researchers.

A similar case of confusion is that of chiastolite, a variety of andalusite (Al_2_SiO_5_), characteristically with carbonaceous inclusions arranged in a way that produces a pattern resembling cross-shaped twinned crystals. However, in this case there are almost no papers confusing the terms.

## 4. Conclusions

Six well established misconceptions related to crystallography and crystal optics have been explained in detail. Taking the motto that a problem is an opportunity in disguise, these misconceptions could be used by teachers as an educational resource. Students pay extra attention when the teacher not only explains a concept but also explains the reasons for its widespread misuse. The attentive student wants to avoid taking part in that misuse, and from that moment on will pay special attention to detect whether any of their classmates or even teachers make the mistake pointed out in class.

The explained misconceptions have been exposed through their recurrent appearances in scientific literature. These will possibly continue to appear in future research papers and lectures, but it is hoped that this paper will contribute to raise awareness among researchers. It is also hoped that these newly aware researchers will comment on the topic in scientific chats, for fun and for the sake of correctness. However, artificial intelligence is becoming increasingly used by both students and academics, and the recurrence of the misconceptions in well credited publications is a source of error in the replies obtained from generative AI tools.

There are a number of reasons for the recurrence of the explained misconceptions, and they often intermingle. The misunderstood cell restrictions (see Section 3.1) possibly arise from the order in which topics are presented in class. Commonly, lattices and unit cells are presented first and only later their associated symmetries, and this produces the false idea that the cell determines the symmetry and not the other way round. Similarity and analogy are also common causes of misconception, *e.g.* trigonal and rhombohedral (see Section 3.2) are often perceived as synonyms and the term spectra (see Section 3.3) is so widely used that its meaning is changing to refer to any result in the form of a plot with peaks or similar features, regardless of the fundamentals of the measuring technique. Under a polarizing microscope in crossed Nicols (XPL), twinning and zoning (see Section 3.6) can appear very similar. Likewise, parallel Nicols (see Section 3.5) seems the ideal complement of crossed Nicols to refer to the two common observational setups of the polarizing microscope. The definition of concepts can sometimes contribute to development of misconceptions, and definitions can vary depending on the context. We have seen this in the case of the misuse of the term alloy (see Section 3.4), since alloy is used in materials science and metallurgy in a sense that infringes the crystallographic sense of alloy. However, definitions can also change over time; for instance, the classical definition of crystal (periodic array of atoms or groups of atoms) was changed in 1992 by the IUCr to encompass both periodic and aperiodic ordered materials (Casas, 2020 ▸). Only authorized institutions can fix or redefine the scientific terminology.

The six cases detailed here are only some examples of misconceptions, and other important but less traceable misconceptions could be cited, *e.g.* many students of crystallography confuse lattice points and atoms/ions, or get the translational equivalence of points confused with the symmetry equivalence of points (Brock & Lingafelter, 1980 ▸). Other instances could also be cited in the field of crystal optics. For example, many students think that birefringence always produces a pair of ordinary and extraordinary rays, and this is not even the general case for biaxial solids (Casas, 2018 ▸). However, many textbooks illustrate the phenomenon with a uniaxial mineral like calcite. Also, it is often assumed that the refraction index correlates with the propagation vector (instead of the vibration vector).

## Figures and Tables

**Table 1:** Summary of the discussed misconceptions

Field	Incorrect uses	Suggested uses	Brief explanation of the misconception
Crystallography (general)	*a* ≠ *b* ≠ *c* and similar inequalities	*a*, *b*, *c* independent	Cell constraints imply only mathematical equalities for some crystal systems.
Crystallography (general)	Trigonal lattice	Rhombohedral lattice	The distinction between trigonal and rhombohedral is relevant to highlight that trigonal space groups can be assigned to either rhombohedral or hexagonal lattice systems.
	Rhombohedral crystal system	Trigonal crystal system	
Crystallography (general)	XRD spectrum	XRD pattern	XRD is not a spectroscopic technique; λ remains unchanged.
Crystallography (inorganic materials)	Ordered solid solution	Intermetallic compound	Solid solutions (monophase alloys) have an inherent crystallographic disorder; if such disorder is absent the material should be simply called a compound.
	Heusler alloys	Heusler compounds	
Crystal optics (general)	Parallel Nicols	PPL	Plane polarized light (PPL) is a setup with a single polarizer below the stage. The parallel Nicols setup implies the use of two polarizers sharing the same polarization direction, which is not commonly used.
Crystal optics (mineral optics)	Hourglass twinning	Hourglass zoning	Twinning is a particular crystal intergrowth including two or more crystal domains. Crystal zoning is a compositional change within a single crystal domain. The two can appear similar under a polarizing microscope.

## Data Availability

The statistical results reported in this article can be accessed upon request.
